# Mapping of a new locus associated with autosomal recessive congenital cataract to chromosome 3q

**Published:** 2010-12-08

**Authors:** Namerah Sabir, S. Amer Riazuddin, Tariq Butt, Farheena Iqbal, Idrees A. Nasir, Ahmad U. Zafar, Zaheeruddin A. Qazi, Nadeem H. Butt, Shaheen N. Khan, Tayyab Husnain, J. Fielding Hejtmancik, Sheikh Riazuddin

**Affiliations:** 1 National Centre of Excellence in Molecular Biology, University of the Punjab, Lahore, Pakistan; 2 The Wilmer Eye Institute, Johns Hopkins University School of Medicine, Baltimore, MD; 3 Layton Rahmatulla Benevolent Trust Hospital Lahore, Pakistan; 4 Allama Iqbal Medical College, University of Health Sciences, Lahore, Pakistan; 5 Ophthalmic Genetics and Visual Function Branch, National Eye Institute, National Institutes of Health, Bethesda, MD

## Abstract

**Purpose:**

To localize the disease interval for autosomal recessive congenital cataracts in a consanguineous Pakistani family.

**Methods:**

All affected individuals underwent detailed ophthalmologic examination. Blood samples were collected and genomic DNA was extracted. A genome-wide scan was performed with fluorescently-labeled microsatellite markers on genomic DNA from affected and unaffected family members and logarithm of odds (LOD) scores were calculated.

**Results:**

Clinical records and ophthalmological examinations suggested that affected individuals have bilateral congenital cataracts. Genome-wide linkage analysis localized the critical interval to chromosome 3q with a maximum LOD score of 3.87 at θ=0; with marker D3S3609. Haplotype analyses refined the critical interval to a 23.39 cM (18.01 Mb) interval on chromosome 3q, flanked by D3S1614 proximally and D3S1262, distally.

**Conclusions:**

Here, we report a new locus for autosomal recessive congenital cataract localized to chromosome 3q in a consanguineous Pakistani family.

## Introduction

Congenital cataracts are one of the major causes of vision loss in children worldwide [1,2]. They can occur in an isolated fashion or as one component of a syndrome affecting multiple tissues [3]. Congenital cataracts can lead to permanent blindness by interfering with the sharp focus of light on the retina, especially during the early developmental periods. Congenital cataracts vary markedly in severity and morphology, affecting the nuclear, cortical, polar, or subcapsular parts of the lens, or in severe cases the entire lens [4].

To-date fourteen loci have been associated with autosomal recessive congenital cataracts [5-18]. Of these loci, mutations in nine genes, Eph-receptor type-A2 (*EPHA2*), connexin50 (*GJA8*), glucosaminyl (N-acetyl) transferase 2 (*GCNT2*), heat-shock transcription factor 4 (*HSF4*), lens intrinsic membrane protein (*LIM2*), beaded filament structural protein 1 (*BFSP1*), alphaA-crystallin (*CRYαA*), betaB1-crystallin (*CRYβB1*), and betaB3-crystallin (*CRYβB3*) have been found [5,7,9,12,14-18].

Here, we report a consanguineous Pakistani family with congenital cataracts. Linkage analysis localized the critical interval to chromosome 3q with a maximum LOD score of 3.87 and haplotype analyses refined the critical interval to a 23.39 cM (18.01 Mb). This is the first report localizing autosomal recessive congenital cataracts to chromosome 3q.

## Methods

### Clinical ascertainment

A total of 125 consanguineous Pakistani families with nonsyndromic cataract were recruited to participate in a collaborative study between the National Centre of Excellence in Molecular Biology, Lahore, Pakistan, and the National Eye Institute, Bethesda, MD, to identify novel loci associated congenital cataracts. Institutional Review Board (IRB) approval was obtained from both Institutes. The participating subjects gave informed consent consistent with the tenets of the Declaration of Helsinki. A detailed medical history was obtained by interviewing family members. Ophthalmic examinations were conducted with slit-lamp microscopy. Approximately 10 ml of blood samples were drawn from affected and unaffected members of the family and stored in 50 ml Sterilin® falcon tubes (BD Biosciences, San Jose, CA) containing 400 μl of 0.5 M EDTA. Blood samples were kept at −20 °C for long- term storage.

### DNA extraction

DNA was extracted by a nonorganic method as described previously [5,10]. Briefly, aliquots of 10 ml blood samples were mixed with 35 ml of TE buffer (10 mM Tris-HCl, 2 mM EDTA, pH 8.0) and the TE-blood mixture was centrifuged at 1,800× g for 20 min. The supernatant was discarded and the pellet was re-suspended in 35 ml of TE buffer and centrifuged at 1,800× g for 20 min. The TE washing was repeated for 2–3 times and the washed pellet was re-suspended in 2 ml of TE. A total of 6.25 ml of protein digestion cocktail (50 μl [10 mg/ml] of proteinase K, 6 ml TNE buffer [10 mM Tris HCl, 2 mM EDTA, 400 mM NaCl], and 200 μl of 10% sodium dodecyl sulfate) was added to the re-suspended pellets and incubated overnight in a shaker (250 rpm) at 37 °C. The digested proteins were precipitated by adding 1 ml of 5 M NaCl, followed by vigorous shaking and chilling on ice for 15 min. The precipitated proteins were pelleted by centrifugation at 1,800× g for 20 min and removed. The supernatant was mixed with equal volumes of phenol/chloroform/isoamyl alcohol (25:24:1) and the aqueous layer containing the genomic DNA was carefully collected. The DNA was precipitated with isopropanol and pelleted by centrifugation at 2,400× g for 15 min. The DNA pellets were washed with 70% ethanol and dissolved in TE buffer. The DNA concentration was determined with a SmartSpec plus Bio-Rad Spectrophotometer (Bio-Rad, Hercules, CA).

### Genotype analysis

A genome-wide scan was performed with 382 highly polymorphic fluorescent markers from the ABI PRISM Linkage Mapping Set MD-10 (Applied Biosystems, Foster City, CA) having an average spacing of 10 cM. Multiplex polymerase chain reaction (PCR) was completed in a GeneAmp PCR System 9700 thermocycler (Applied Biosystems). Briefly, each reaction was performed in a 5 μl mixture containing 40 ng genomic DNA, various combinations of 10 mM dye-labeled primer pairs, 0.5 ml 10× GeneAmp PCR Buffer (Applied Biosystems), 1 mM dNTP mix, 2.5 mM MgCl2, and 0.2 U Taq DNA polymerase (Applied Biosystems). Initial denaturation was performed for 5 min at 95 °C, followed by 10 cycles of 15 s at 94 °C, 15 s at 55 °C, and 30 s at 72 °C and then 20 cycles of 15 s at 89 °C, 15 s at 55 °C, and 30 s at 72 °C. The final extension was performed for 10 min at 72 °C. PCR products from each DNA sample were pooled and mixed with a loading cocktail containing HD-400 size standards (Applied Biosystems). The resulting PCR products were separated in an ABI 3100 DNA Analyzer (Applied Biosystems) and genotypes were assigned with GeneMapper software (Applied Biosystems).

### Linkage analysis

Two-point linkage analyses were performed using the FASTLINK version of MLINK from the LINKAGE Program Package, whereas the Maximum LOD scores were calculated with ILINK from the LINKAGE Program Package [19,20]. Autosomal recessive cataract was analyzed as a fully penetrant trait with an affected allele frequency of 0.001. The marker order and distances between the markers were obtained from the Marshfield database and the National Center for Biotechnology Information (NCBI) chromosome 3 sequence maps. For the initial genome scan, equal allele frequencies were assumed, while for fine mapping allele frequencies were estimated from 96 unrelated and unaffected individuals from the Punjab province of Pakistan.

## Results

**Figure 1:**
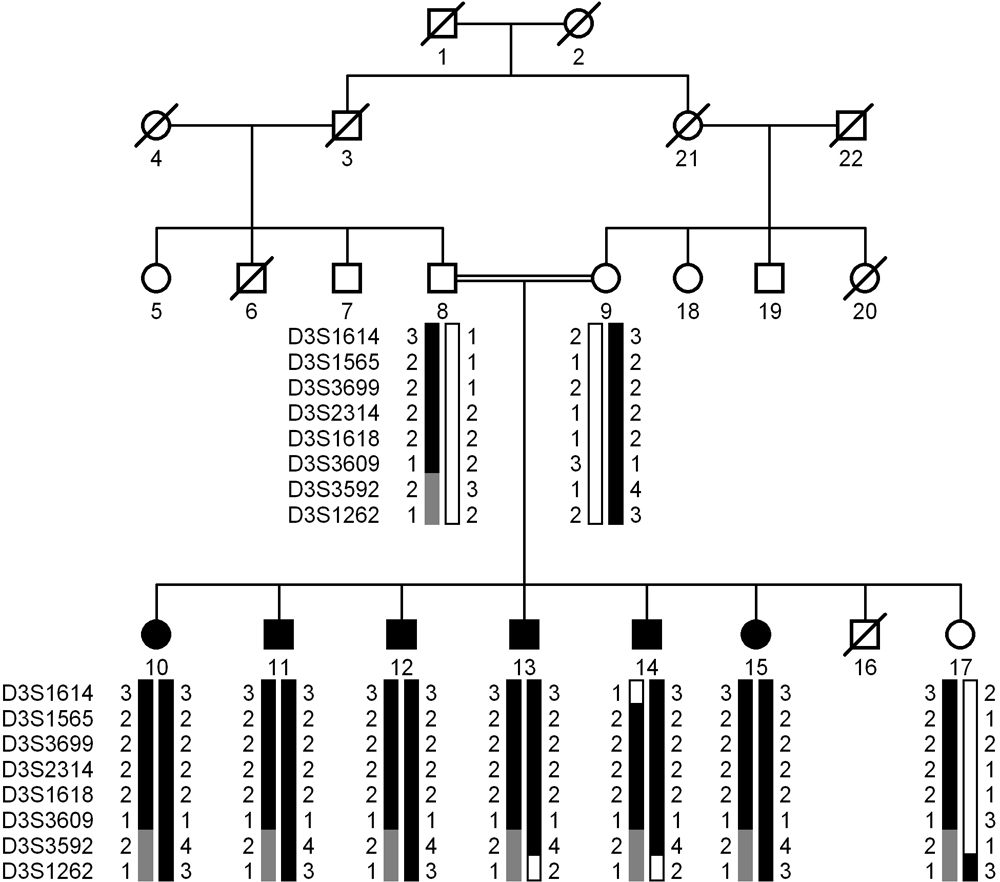
Pedigree drawing and haplotypes of chromosome 3q markers of family PKCC144. The haplotypes of 8 adjacent chromosome 3q microsatellite markers are shown with alleles forming the risk haplotype are shaded black, alleles co-segregating with cataracts but not showing homozygosity are shaded gray and alleles not co-segregating with cataracts are shown in white. Squares are males, circles are females, and filled symbols are affected individuals; the double line between individuals indicates consanguinity and the diagonal line through a symbol is a deceased family member.

A large consanguineous family, PKCC144 consisting of six affected and three unaffected individuals was recruited from the Punjab province of Pakistan (Figure 1). A detailed medical and family history was obtained from all affected and unaffected members of family. The clinical records available to us were suggestive of bi-lateral congenital cataracts with no other ocular or systemic abnormalities present in the family (Table 1). As all the affected individuals have had cataract surgery prior to the enrollment, therefore no photographic evidence of cataract was available to classify the cataracts.

**Table 1:** Clinical characteristics of the affected individuals of family PKCC144.

**Individual number**	**Age at recruitment (years)**	**Age of onset**	**Classification of the cataract**	**Extra ocular anomalies**
10	18	Congenital	Unknown	None
11	15	Congenital	Unknown	None
12	13	Congenital	Unknown	None
13	12	Congenital	Unknown	None
14	5	Congenital	Unknown	None
15	4	Congenital	Unknown	None

Initially, linkage to all reported loci associated with autosomal recessive cataract loci were excluded by haplotype analyses with closely spaced fluorescently-labeled microsatellite markers (data not shown). Subsequently, a genome-wide linkage scan was completed and a maximum two-point LOD score of 3.87 at θ=0 was obtained with D3S3609 during the genome-wide scan (Table 2). Additional STR markers were selected to further analyze the critical interval, which further provided evidence for linkage to chromosome 3q with LOD scores of 3.42, 2.02, 2.33, and 2.04, at θ=0 with markers D3S1565, D3S3699, D3S2314, and D3S1618, respectively (Table 2).

**Table 2:** Two-point LOD scores of chromosome 3q markers.

**Markers**	**cM**	**Mb**	**0**	**0.01**	**0.05**	**0.09**	**0.1**	**0.2**	**0.3**	**Z_max_**	**θ_max_**
D3S1614	177.75	168.21	-∞	−0.17	0.41	0.52	0.54	0.51	0.36	0.54	0.10
D3S1565	186.04	173.48	3.42	3.35	3.02	2.7	2.61	1.8	1.06	3.42	0.00
D3S3699	191.79	179.29	2.02	1.99	1.83	1.68	1.64	1.23	0.81	2.03	0.00
D3S2314	193.75	182.13	2.33	2.28	2.1	1.92	1.88	1.4	0.92	2.33	0.00
D3S1618	194.68	183.35	2.04	2.01	1.85	1.68	1.64	1.23	0.8	2.04	0.00
D3S3609*	195.60	184.03	3.87	3.8	3.51	3.22	3.14	2.37	1.55	3.87	0.00
D3S3592	198.68	184.44	0.23	1.8	2.23	2.21	2.19	1.75	1.13	2.23	0.05
D3S1262*	201.14	186.22	-∞	−4.13	−1.56	−0.76	−0.63	−0.01	0.05	0.05	0.30

The asterisk indicates markers used in the genome-wide scan.

Visual inspection of the haplotypes supported the results of linkage analysis (Figure 1). There is a recombination in affected individual 14 and D3S1614 that defines the proximal boundary (Figure 1). Similarly, recombination events in affected individuals 13 and 14 at D3S1262 define the distal boundary (Figure 1). This places the pathogenic locus in a 23.39 cM (18.01 Mb) interval. Additionally, a lack of homozygosity in affected individuals 10, 11, 12, 13, 14, and 15 at D3S3592 further suggests that the pathogenic mutations lies proximal to D3S3592, in a 20.93 cM (16.23 Mb) interval flanked by D3S1614 proximally and D3S3592 distally.

## Discussion

Here, we report a new locus for autosomal recessive congenital cataracts localized to chromosome 3q, in a consanguineous Pakistani family. Genome-wide linkage analyses localized the critical interval to chromosome 3q with significant LOD scores and these results were further supported by haplotype analyses, which places the critical interval 23.39 cM (18.01 Mb) flanked by markers, D3S1614 proximally and D3S1262 distally. Additionally, lack of any significant Lod scores obtained during the genome-wide scan other than with marker D3S3609, further supports linkage to chromosome 3q. This is the first report associating chromosome 3q with autosomal recessive congenital cataracts.

The critical interval is a gene rich region that harbors more than 100 genes according to the UCSC database. Subsequent to prioritizing the genes present in the critical interval based on their known or proposed function and expression especially in the lens, we are sequencing coding exons of these candidate genes in one affected and one unaffected individual. Identification of the gene responsible for recessive congenital cataract is expected not only to provide insight and clues to the allied clinical findings but it will also increase our understanding of molecular events involved in the lens biology.
